# Recurrent hypoglycaemia in type 2 diabetic patient due to hypothyroidism

**DOI:** 10.1186/s40200-015-0149-y

**Published:** 2015-03-19

**Authors:** Alok Kumar

**Affiliations:** MK Diabetes Clinic, India, SB-132, Shastri Nagar, Ghaziabad, 201002 UP India

**Keywords:** Hypoglycaemia, Type 2 diabetes mellitus, Hypothyroidism, Thyroid

## Abstract

**Objective:**

To present a case report of recurrent hypoglycaemia in type 2 diabetic patient due to hypothyroidism.

**Methods:**

We describe a case with type 2 diabetes who complained of episodes of recurrent hypoglycaemia in last few weeks. We also discuss the results of the diagnostic workup done to find out the cause of recurrent hypoglycaemia.

**Result:**

After ruling out common causes like overdosing of oral hypoglycaemic agents or insulin, renal or hepatic impairment we found overt hypothyroidism as a cause of recurrent hypoglycaemia. Levothyroxine therapy was started in this patient. Upon normalization of thyroid function, patient did not further experience any episodes of hypoglycaemia. We did not find any report on hypothyroidism as a cause of recurrent hypoglycaemia in type 2 diabetic patients.

**Conclusion:**

We conclude that during workup, clinicians should take hypothyroidism into consideration as a potential cause of hypoglycaemia in type 2 diabetic patients.

## Background

Hypoglycaemia is a common condition in diabetic patients. Prevalence of hypoglycaemia has been observed in 12% of patients on diet alone followed by 16% and 30% in patients treated with oral hypoglycaemic agents and insulin respectively [1]. Contrary to the belief that hypoglycaemia is less common in type 2 diabetes, in a study by Kasia et al. 9.3 - 13.8% type 2 diabetic patients experienced hypoglycaemia irrespective of glycaemic control whereas 10.8% patients experienced severe hypoglycaemia [2]. The most common causes of hypoglycaemia in diabetic patients are: overdosing of oral hypoglycaemic agents or insulin with respect to meal and physical activity, renal or hepatic impairment [3]. In hypothyroidism gastrointestinal absorption of glucose and its utilization in the peripheral tissues is slowed [4-6]. Hepatic glucose output is decreased and insulin half-life is prolonged [4-6]. Doses of exogenous insulin may have to be decreased to avoid hypoglycaemia [5,6]. However, after treatment with levothyroxine when thyroid function is normalized blood glucose levels may again increase to pre-treatment levels [4-6]. Hypothyroidism is not considered as a most common cause of hypoglycaemia in a type 2 diabetic patient. Here we present a case of recurrent hypoglycaemia in a type 2 diabetic patient diagnosed with overt hypothyroidism.

## Case presentation

In February 2014 a 73 years old male with type 2 diabetes for last 20 years visited our outpatient clinic. He complained of episodes of recurrent hypoglycaemia in last few weeks. His most recent blood glucose values in his record book included many values of less than 70 mg/dl. Two days prior to his clinic visit patient experienced a severe episode of hypoglycaemia with blood glucose value of 39 mg/dl from which he later recovered by taking fast acting carbohydrates. During initial years diabetes was managed with oral hypoglycaemic agents [OHA’s]. After twelve years of OHA therapy, once daily 8 units of insulin glargine was added in the year 2006, subsequently sulphonylurea and dipeptidyl peptidase – 4 [DPP-4] inhibitor were stopped. His glycemic control remained satisfactory in the following years. Based on his elevated HbA1C (11.2%) and results of home blood glucose monitoring, in the year 2011, a premeal insulin glulisine 8 units before each meal was started along with insulin glargine 10 units once a day and metformin 500 mg twice daily. In the past two years his glycosylated haemoglobin [HbA1C] ranged 7.9% to 9.4% and his insulin doses almost remained the same.

On physical examination his body mass index [BMI] was 22.72 kg/m2, blood pressure was 114/82 mmHg, pulse rate was regular at 62 beats/ minute. No significant change was noticed in the parameters during his past follow up visits. His latest retinal examination in January 2013 showed no evidence of diabetic retinopathy.

Further, based on his blood glucose monitoring, doses of insulin glulisine were reduced to 4 units before each meal and to 6 unit of insulin glargine once daily. Preliminary biochemical investigations were done to find out the cause of recurrent hypoglycaemia. Results of biochemical results showed HbA1c - 7%, Normal renal parameters (urinary albumin to creatinine ratio = 5.04 mg/g (normal < 30 mg/gm creatinine, serum creatinine was 0.85 mg/dl), serum alanine aminotransferase (SGPT) was also in normal range 21 U/L (ref. Range 10–65 U/L), Free thyroid function test revealed raised serum thyroid stimulating hormone [TSH] ( 6.121 uIU/ml, ref. Range 0.5 – 4.780 uIU/ml ultrasensitive assay), low free T4 – 0.83 ng/dl (ref. Range 0.89 – 1.76 ng/dl) and normal free T3 – 2.76 pg/ml (ref. range 2.3-4.2 pg/ml). TPO antibodies were negative. Diagnosis of overt hypothyroidism was made and patient started levothyroxine therapy. Six weeks later his thyroid function normalized. Serum TSH was 3.733 uIU/ml (ref. Range 0.5 – 4.780 uIU/ml ultrasensitive assay) and serum Free T4 was 1.47 ng/dl (ref. Range 0.89 – 1.76 ng/dl). This patient did not have past history of thyroid dysfunction and three years back his serum TSH was 3.4 uIU/ml. After normalization of thyroid function patient did not experience any episode of hypoglycaemia, although based on his self glucose monitoring values, insulin doses were again increased to the same doses that patient was taking earlier.

## Discussion

Hypoglycaemia is usually seen in type 2 diabetic patients who take sulphonylureas and insulin. In the case described here we ruled out any change in patient’s recent schedule, increase in physical activity, eating habits and anti diabetic treatment induced hypoglycaemia. There was no history of any drug interaction. Renal or hepatic insufficiency was excluded. Although hypothyroidism is not considered as one of the most common cause of hypoglycaemia in routine clinical practice, in literature there is a mention that hypothyroidism may cause hypoglycaemia uncommonly [7]. In hypothyroidism, important factors that contribute to hypoglycaemia are reduced insulin clearance, slow gastric emptying and decreased intestinal absorption of glucose. Further, biochemical effects like reduced gluconeogeneis, impaired glycogenolysis and reduced glucagon secretion prevents recovery from hypoglycaemia [6,8].

The prevalence of thyroid dysfunction in type 2 diabetics has been reported to be high [8-10]. After assessing the thyroid function test diagnosis of overt hypothyroidism was made because of the raised TSH and low free T4. Patient was started on levothyroxine therapy and further investigations were avoided due to the economic constraint. Upon normalisation of thyroid function in the last one month and later during subsequent follow up clinic visits for next six months patient did not report any episodes of hypoglycaemia. Due to raised glucose levels gradually insulin doses were increased almost equal to the same doses which patient was taking earlier.

Incidence of hypothyroidism in type 1 diabetic patients is high and these patients are usually screened for thyroid dysfunction due to the autoimmune link. We did not find any report on hypothyroidism as a cause of recurrent hypoglycaemia in type 2 diabetic patients.

## Conclusion

In conclusion, we suggest that during diagnostic workup clinicians should take hypothyroidism into consideration as a potential cause of recurrent hypoglycaemia in type 2 diabetic patients.

## Consent

“Written informed consent was obtained from the patient for publication of this Case report and any accompanying images. A copy of the written consent is available for review by the Editor-in-Chief of this journal”.
